# A conversation on the effects of the COVID-19 pandemic on academic careers with junior researchers

**DOI:** 10.1038/s41467-021-22039-w

**Published:** 2021-04-07

## Abstract

The various restrictions applied across the globe to contain the COVID-19 pandemic have been impacting the way we knew how to work. Ms. Wilson (a PhD student in Earth System Science at Stanford University), Dr. Xin (a glia biologist and postdoctoral fellow at University of California San Francisco), and Dr. Saidaminov (a researcher in advanced functional materials and Assistant Professor at the University of Victoria) shared with *Nature Communications* their thoughts on how the COVID-19 pandemic is affecting their professional development and career progression and their coping strategies.

Tell us a bit about your research background and position.

I’m **Alexis Wilson** (she/her), a second year PhD student in the department of Earth System Science at Stanford University. I study the intersection of soil biogeochemistry, climate change, and environmental justice. My passion lies in understanding and combating environmental racism and climate injustices on local and global scales. My current research focuses on assessing soil contamination in urban gardens, particularly in marginalized communities in the San Francisco Bay Area. I aim to work in collaboration with community organizations to prioritize and address community identified needs through my work.

**Figure:**
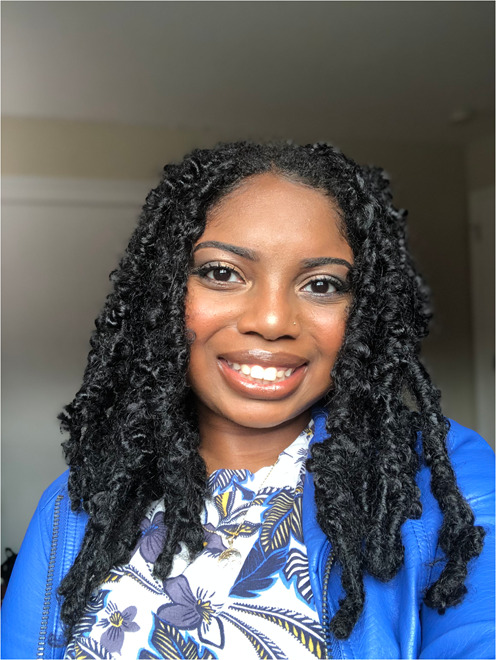
Alexis Wilson, PhD student, Stanford University.

I’m **Wendy Xin**. I received a PhD in Neuroscience from Johns Hopkins University and have been a postdoctoral fellow at the University of California, San Francisco (UCSF) since March 2019. I study oligodendrocytes; these cells make myelin in the central nervous system. Oligodendrocytes and myelin are formed and reshaped by sensory experiences and learning throughout life, and these changes appear to be critical for preserving long-term memory. My research is focused on understanding how plasticity within the oligodendrocyte lineage alters neuronal circuit function in development, learning, and disease.

**Figure:**
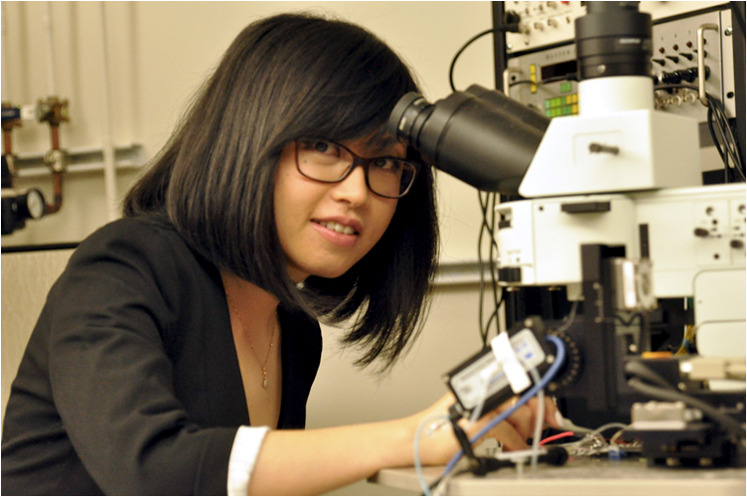
Wendy Xin, postdoctoral researchers, University of California San Francisco. Daniel Pham

I am **Makhsud Saidaminov**. I’m a Canada Research Chair in Advanced Functional Materials and an Assistant Professor at the University of Victoria. Our research team’s mission is to enable a green economy. We understand our responsibility for the climate crisis and its key driver—the carbon economy. To mitigate this challenge, we develop new materials and technologies to harvest and consume solar energy efficiently.

**Figure:**
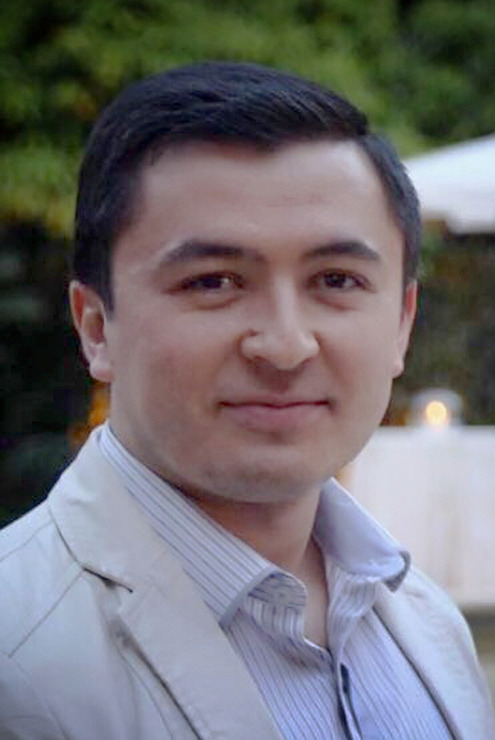
Makhsud Saidaminov, Assistant Professor, University of Victoria.

What are you doing to cope or counter any COVID-19-related difficulties you have faced so far?

**AW:** I was finishing my first year of graduate school when the pandemic began and forced us to complete the rest of the academic year remotely. We are currently attending classes and conducting research remotely. Transitioning to remote learning and research has been very difficult but I’ve tried to adjust as best as possible. Some strategies I’ve utilized to fulfill my academic duties are lowering my workload where possible, adjust my research focus to work that can be done online vs. in the lab, and make reasonable/attainable goals considering the situation. To cope mentally, I connect with people virtually, enjoy the outdoors when possible, and do some of my favorite activities, like reading and painting. During this time, I think it’s important to balance work and relaxation/socializing as much as possible.

**WX:** On the macro scale, I feel incredibly lucky and grateful to have a job and a place to live, which so many have lost. Science-wise, my university shut down for around two months, then moved to a gradual re-opening (25% of regular occupancy) in June. I haven’t come up with any great coping strategies on that front, besides regularly reminding myself that we are all going through this strange experience together and that ruminating over the career ramifications won’t change this reality. One positive side effect of the pandemic is the now widespread use of video conferencing platforms for casual interactions. I have connected with quite a few trainees who reached out to me via email or Twitter to talk about graduate school applications and postdoc searches. These opportunities to be helpful to someone else have been a small reprieve in what is otherwise a deeply isolating time.

**MS:** Shutting down research labs disproportionately affected young faculty. Building the lab, training first group members, and collecting preliminary data for grants cannot be done online. As a new faculty with expertise and a plan for experimental research only, I had to reshape my plan and adapt due to the lockdown. I decided to be a student again, this time to learn the basics of computational science. I finally found time for this, though it was waiting on my to-do list for some time. It was a rewarding experience—the gained knowledge and generated data helped to write grant applications, reviews and perspective papers, and even informed our experimental plan.

What do you think are the likely short and/or long-term consequences for future project funding and academic career progression due to the pandemic?

**AW:** I don’t think it’s possible that the pandemic will have no effect on future project funding or academic career progression. There is bound to be some impact, however, I can’t make this prediction as I am still in an early phase in my career and learning more about these processes each day. I do hope that the impacts of the pandemic are considered when making future decisions and people aren’t penalized for things that may have been out of their control and thus impacted their work during this time.

**WX:** In terms of funding availability, I guess it will depend on how quickly each country can get COVID-19 under control and begin the process of economic recovery, as well as how much the government prioritizes funding a similar portfolio of basic science when there are so many fires to put out. I am not expecting any significant increases in funding into basic neuroscience in the near future. As for career progression, I fear (as perhaps all postdocs do) there will be an accumulation of highly qualified candidates flooding an already saturated and maybe shrinking academic job market, due to undergraduate/medical institutions losing income from tuition, elective medical procedures, donations, etc. I also worry for more senior colleagues who can’t afford to put off job searches for one or more cycles and will be forced to stop pursuing their ideal positions due to financial or immigration status limitations. I’m particularly concerned for my colleagues with children, especially mothers, who are overwhelmed with childcare duties and who have thus far only seen commiserating opinion articles, but no concrete institutional support.

**MS:** Research is mostly an evolutionary process: knowledge is gained and sharpened on a day-to-day basis. But gaps often reset the research progress. This means that the delay in researchers’ career progression will be longer than the actual time of lockdown. However, I remain optimistic about the future of science post-pandemic. Some funding agencies have increased support of research related to COVID-19. If the pandemic taught us one lesson, it is that science had to be supported, trusted, and relied upon. I want to believe that this enthusiasm for increased support of science will remain post-pandemic.

In your opinion, what can supervisors, mentors, and/or university leadership do to support early career researchers (ECRs) and junior faculty during lockdowns and post-pandemic?

**AW:** I think it’s important that supervisors, mentors, professors, etc. have a conversation with their students and adjust the expectations and goals of the ECRs. My advisors worked with me to adjust my research goals so that I can still make progress on my degree remotely. Supervisors should provide professional support during this time (helping us stay on track in our careers) and also check-in /foster community within the lab group. Institutionally, administration should offer support structures (academic, personal, and financial) for their students and staff. This could be funding support, access to COVID-19 testing and supplies (masks), or mental health support. Post-pandemic I think it will be important to re-adjust expectations and have a clear understanding about how things have changed and how that might compare to the way things were structured pre-pandemic. I also think it will be important to keep those support structures in place because the impacts of the pandemic may be long-lasting (mental health, financial, and professional support).

**WX:** Supervisors should proactively, and regularly, check in on the well-being and needs of their trainees. Many trainees don’t want to burden their advisors during a universally challenging time, but probably would want extra guidance, or even just clarity, on the expectations of their advisors for their productivity and training under these circumstances. In particular, supervisors could help trainees who have dependents, or other circumstances that make it challenging to work in the lab, find collaborators within and outside the lab to help lessen their experimental burden and push their projects forward. These conversations/interactions tend to be easier for supervisors to initiate. Institutions should explicitly change their tenure requirements and/or timelines to help relieve the burden felt by pre-tenured faculty who (1) frequently have family obligations and (2) want to avoid pressuring their trainees into working under unsafe conditions.

**MS:** Networking is essential for young faculty. In pre-pandemic times, running into new people at conferences or discussing random topics with colleagues on university hallways helped new faculty to adapt to a new environment and broaden their network. In pandemic time, when meetings occur online, this element of spontaneity is absent. Online meetings are generally organized with a clear agenda for discussion. I encourage young faculties to be proactive in online events and not to be shy to ask for help if they need it, and university administrators to facilitate such interactions.

This pandemic will end, and universities will resume full-time operation, which will likely lead to high demand for Highly Qualified Personnel, laboratory instrumentation, and supplies. Young faculties need support at this stage as they build their research and make some of the most important decisions in their career. I hope that we will not be back to normal, but rather forward to a better post-pandemic academia.

Do you think the pandemic is exacerbating/will exacerbate issues associated with diversity and inclusion in academia? If so, in what way? In your opinion, what can be done to prevent this?

**AW:** If leaders in academia do not address the ways the pandemic (along with other social crises happening at the moment) is specifically impacting marginalized groups, it could further isolate and distress these groups. Offering words of support is not enough; students should be heard by administration and supported by concrete actions. Additionally, the application season for graduate school and/or faculty positions may be impacted by the pandemic. Some departments have responded by removing the GRE [Graduate Record Examination] requirement for this year. Applicants this year and in the future should not be penalized for the ways the pandemic may have impacted their academics during this time. Not taking this into consideration could result in qualified candidates from diverse backgrounds being excluded from consideration. Overall, academia should not try “one-size fits all” solutions and instead address the specific needs of each group, particularly underrepresented groups.

**WX:** Without a doubt. In the first months of the current pandemic, there has been a drop of preprint submissions where at least one of the leading (first or last) authors was a woman. This ongoing study seems to suggest that preprint submissions from women in STEM are getting better. Nevertheless, these observations highlight that the COVID-19 pandemic did not affect all researchers equally.

Perhaps less obvious is the strain the pandemic puts on individuals with extra financial burdens and no alternative sources of income. Academics who cannot rely on savings or family support may be struggling to cover increased costs associated with the pandemic (e.g. shouldering the cost of maintaining a vehicle, instead of using public transit) or need to financially support other family members during this period. These external financial pressures will make it increasingly difficult for people from disadvantaged backgrounds to stay in academia, where pay is limited at the graduate student and postdoc levels. There are no easy solutions to these pervasive inequities that have been amplified by the pandemic. Universities could offer grants to support those who have incurred additional costs due to the pandemic and arrange access to safe and affordable childcare. On the positive side, the large number of virtual conferences and symposia has made scientific exchange more inclusive. We should continue hosting high-quality, high-participation virtual conferences post-pandemic to maintain accessibility and reduce our collective environmental impact.

**MS:** Competition for a faculty position is tough: hundreds of applications are usually received for each academic job opening. The pandemic is projected to cut significant jobs in the university sector. This increased competition may further exacerbate issues of diversity and inclusion in academia. We should work to save and even increase jobs in academia. And we have to hold all our commitments for equity and diversity in academia, no matter what. There should not be a compromise for fairness in academia.

Facing the unexpected challenges of the pandemic, have you or your lab mates come up with any creative or innovative solutions to continue your research and maintain/establish collaborations?

**AW:** My lab groups continue to have weekly lab meetings to update each other on research progress and gather feedback. We use this space to welcome guest presenters and speakers on various topics. We have informal online spaces where we can meet to discuss life outside of research. We are also incorporating group social activities into our meetings so that we can continue to build and foster community within the group. During this time, we are all doing our best to stay connected and support each other professionally and personally, whether that entails sharing resources on a new technique or sharing a new baking recipe we tried that weekend.

**WX:** Like most other labs, we have moved all in-person interactions to Zoom. We have a group chat for people to ask questions or lab favors. Nevertheless, collaborations have become more challenging, as everyone’s schedules are less flexible now due to restrictions in density in indoor spaces, like the lab, and additional limitations in our personal lives. On the other hand, everyone has become more adept at Zoom, which has elevated the quality of digital interactions that might have been more stilted in the pre-pandemic world. Perhaps this change in culture will actually lower the threshold for initiating cross-institutional collaborations, as within-institution collaborations no longer have the advantage of casual in-person interactions.

**MS:** Our team is leaning toward the automation of research. This reduces human physical involvement and accelerates systematic studies. In the context of our field, this leads to the accelerated discovery of new materials. But I have to admit that I miss the close interaction with my students. I witnessed multiple occasions when discoveries are noticed, or the best ideas are born only during face-to-face brainstorming, right on top of the experimental setup. To ensure that we don’t miss those moments, we use communication hubs to instantaneously exchange knowledge, observations, and feedback.

In terms of collaborations, we indeed feel that partnerships involving experimental research have slowed down due to limited access to research labs. Nevertheless, we united with several other groups to write perspective and review papers.

Any other thoughts?

**AW:** For decision makers, it is important to realize that not everyone has access to the same amount of information and resources necessary to survive this pandemic and to excel academically and professionally. For example, access to stable internet and technology, family support (e.g., child-care), and resources on how to best be successful when working remotely. I have never had to work online, I’ve always had a structured in-person learning experience, so information and resources on how to successfully work remotely would have been helpful. Instead I’ve been piecing it together on my own and doing my best to adapt. In summary, providing genuine, tangible support, and being patient and understanding during this time is really needed to support ECRs.

**WX:** I have seen many senior scientists express concern for ECRs struggling to juggle family obligations with their professional ones. There is a general acknowledgement that this struggle will lead to lower productivity during this period. However, I am not confident the empathetic tone struck during these discussions will translate to consideration in decision-making committees. I would urge grant and tenure committees to actually stand up for junior colleagues whose productivity suffered because they needed to attend to their families and/or gave their trainees leeway to do the same, rather than feel relieved of this duty because they paid the issue sufficient lip service on Twitter.

**MS:** One silver lining of the pandemic is that most conferences moved to the online format. Online conferences are more inclusive, allowing attendees to join from anywhere in the world with little or no fees. It also saves time, stress, and resources associated with physical attendance. I gave five online invited talks this year—this would not be possible by physical attendance, especially at my career stage. The pandemic also gave us a chance to think about our core values, such as family, work-life balance, connections. In the first days of lockdown, I reunited virtually with my previous colleagues, who are also young faculties today. We meet regularly to share our experiences and strategies to navigate through the pandemic. One lesson I learned from these meetings is that we are not alone, and we should stay strong and connected.

